# A novel restriction endonuclease GlaI for rapid and highly sensitive detection of DNA methylation coupled with isothermal exponential amplification reaction†
†Electronic supplementary information (ESI) available. See DOI: 10.1039/c7sc04975g


**DOI:** 10.1039/c7sc04975g

**Published:** 2017-12-11

**Authors:** Yueying Sun, Yuanyuan Sun, Weimin Tian, Chenghui Liu, Kejian Gao, Zhengping Li

**Affiliations:** a Key Laboratory of Applied Surface and Colloid Chemistry , Ministry of Education , Key Laboratory of Analytical Chemistry for Life Science of Shaanxi Province , School of Chemistry and Chemical Engineering , Shaanxi Normal University , Xi’an 710062 , Shaanxi Province , P. R. China . Email: liuch@snnu.edu.cn ; Email: lzpbd@snnu.edu.cn

## Abstract

Sensitive and accurate detection of site-specific DNA methylation is of critical significance for early diagnosis of human diseases, especially cancers. Herein, for the first time we employ a novel methylation-dependent restriction endonuclease GlaI to detect site-specific DNA methylation in a highly specific and sensitive way by coupling with isothermal exponential amplification reaction (EXPAR). GlaI can only cut the methylated target site with excellent selectivity but leave the unmethylated DNA intact. Then the newly exposed end fragments of methylated DNA can trigger EXPAR for highly efficient signal amplification while the intact unmethylated DNA will not initiate EXPAR at all. As such, only the methylated DNA is quantitatively and faithfully reflected by the real-time fluorescence signal of the GlaI–EXPAR system, and the potential false positive interference from unmethylated DNA can be effectively eliminated. Therefore, by integrating the unique features of GlaI for highly specific methylation discrimination and EXPAR for rapid and powerful signal amplification, the elegant GlaI–EXPAR assay allows the direct quantification of methylated DNA with ultrahigh sensitivity and accuracy. The detection limit of methylated DNA target has been pushed down to the aM level and the whole detection process of GlaI–EXPAR can be accomplished within a short time of 2 h. More importantly, ultrahigh specificity is achieved and as low as 0.01% methylated DNA can be clearly identified in the presence of a large excess of unmethylated DNA. This GlaI–EXPAR is also demonstrated to be capable of determining site-specific DNA methylations in real genomic DNA samples. Sharing the distinct advantages of ultrahigh sensitivity, outstanding specificity and facile operation, this new GlaI–EXPAR strategy may provide a robust and reliable platform for the detection of site-specific DNA methylations with low abundances.

## Abstract 2

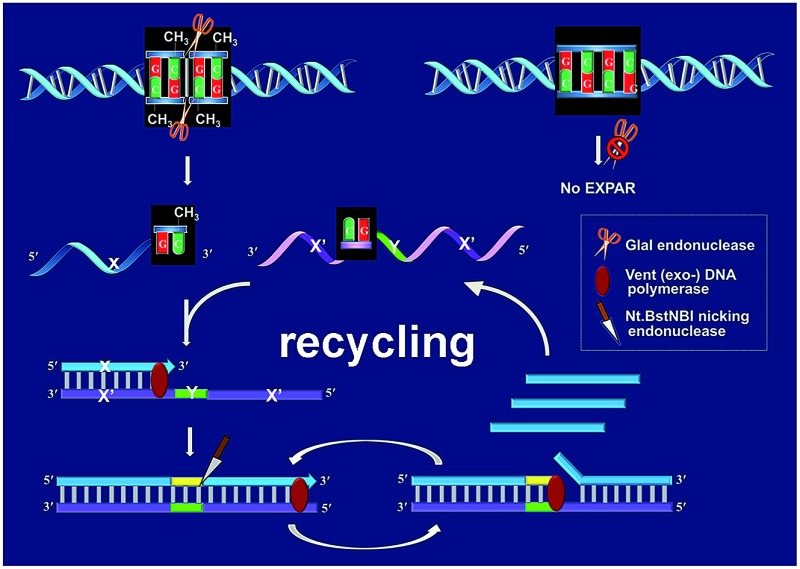
An elegant GlaI–EXPAR strategy is proposed which allows accurate detection of site-specific DNA methylations with ultrahigh sensitivity and specificity.

## Introduction

DNA methylation at the C5 position of cytosine in CpG dinucleotides is the most frequent and important epigenetic modification mechanism in mammalian cells, which plays critical roles in the regulation of gene expression, genomic imprinting, and X-chromosome inactivation.^1^,^2^ Aberrant CpG methylation states, especially those that occur in the gene promoter regions, have been proved to be closely associated with the development of many human diseases including cancers.^3^ The CpG hypomethylation in the promoter of oncogenes or the hypermethylation in the promoter of tumor-suppressor, have been suggested to be crucial drivers for cancer progression.^4^ So the alterations of DNA methylation may serve as important biomarkers for early diagnosis, classification as well as prognosis of cancers.^5^–^7^ In this regard, the highly sensitive and accurate quantification of site-specific DNA methylation will be of great significance for biomedical studies.^8^

Generally, in real clinical samples, the tumor-derived methylated DNA is only present in rather low quantities (less than 0.1%) with the majority of the DNA being derived from normal cells.^9^ So how to effectively discriminate DNA target with a specific methylation site from a large pool of unmethylated DNA is the most critical issue for the detection of DNA methylation. According to the employed methylation discrimination mechanisms, the conventional methylation assays can be mainly classified into two categories, namely, the bisulfite conversion (BC)-assisted approaches and the methylation-sensitive restriction endonuclease (MSRE) digestion-based protocols. BC is a chemical method that can convert cytosines (C) into uracils (U) but leave the methylated cytosines unchanged with the treatment of sodium bisulfite.^3^,^10^,^11^ In such cases, the detection of methylated cytosine sites from the unmethylated counterparts can be fulfilled by discriminating the resulting base differences (methylated cytosine *vs.* uracil) between the DNA targets. Although widely applied as the gold-standard methylation assays, the BC-assisted protocols suffer heavily from cumbersome and time-consuming procedures, which need multiple steps including target denaturation with NaOH, treatment with bisulfite, desulfonation process and DNA purification under stringent experimental conditions. More seriously, the BC-based chemical treatment of DNA targets will lead to inevitable DNA degradation, incomplete conversion as well as DNA loss during the multi-step operations, which may result in batch-to-batch inconsistency and finally misleading results for methylation analysis,^12^ particularly for the targets with very low levels of methylation.

MSREs (*e.g.*, HpaII, AciI and BsoFI) are a group of enzymes that can identify and cleave DNA at the unmethylated cytosine site while the methylated DNA will not be digested and thus remain intact.^13^ The MSRE digestion-based methylation assays are typically based on the determination of uncleaved methylated DNA by using appropriate DNA amplification strategies with the assumption that the unmethylated DNA is completely cleaved and thus cannot be amplified.^14^–^17^ Compared with BC treatment, the MSRE digestion-based methylation assays are performed under mild conditions with rapid and simple operation. Nevertheless, since the methylated DNA targets only constitute a very small percentage of the overall DNA pool in most cases, even a trivial portion of incomplete cutting of the unmethylated DNA will lead to significant false positive interference for the quantification of the low-abundance methylated targets.

Totally different from MSREs, GlaI, a newly discovered methylation-dependent DNA restriction endonuclease, can specifically recognize and cleave DNA with methylated cytosines while cannot cut DNA with unmethylated cytosines.^18^,^19^ Inspired by the fact that the GlaI enzyme exhibits ultrahigh specificity towards the digestion of methylated DNAs with simple operation, we believe that if the GlaI-cleaved fragments of methylated DNA can be specifically and rapidly determined against the undigested DNAs, the low-abundance DNA methylation can be more faithfully quantified with high accuracy and reliability even when the unmethylated DNAs outnumber it massively. Based on this premise, herein, we wish to develop an elegant GlaI-assisted DNA methylation assay with high sensitivity, specificity and accuracy.

In this regard, besides for the GlaI-assisted specific discrimination between methylated and unmethylated cytosines in DNA targets, how to achieve high sensitivity is another key issue for the detection of rare DNA methylation in limited genomic samples. In order to obtain enough sensitivity for methylation analysis, DNA amplification protocols are generally required by coupling with either BC treatment or restriction endonuclease digestion.^14^,^20^,^21^ Among them, the various adapted polymerase chain reaction (PCR) protocols, such as the methylation specific PCR,^22^ PCR-based single-nucleotide primer extension,^23^ end-specific PCR (ESPCR)^24^ and so on, are the most widely employed methods for DNA methylation analysis. However, the PCR-based methods have some inevitable limitations. First, specific PCR relies heavily on the stringent primer/template design and precisely optimized thermal cycling. If not fully optimized, the frequent false-positive results of PCR may lead to uncertain results. Second, most of the PCR-based methylation assays need to be combined with further post-PCR detection steps or even additional signal amplification routes, making the assay procedures rather complicated. As an alternative of PCR, ligase chain reaction (LCR) has been developed recently by our group and others for site-specific methylation analysis with high sensitivity and specificity,^25^–^27^ but precisely controlled thermal cycles similar to PCR are still indispensable. To tackle such drawbacks associated with the thermal cycling PCR and LCR, several isothermal amplification methods, such as rolling circle amplification (RCA)^28^–^30^ and strand displacement amplification (SDA),^31^,^32^ are proposed to detect DNA methylation under isothermal conditions, which effectively avoid the optimization of thermal cycles. Nevertheless, both RCA and SDA need long reaction time up to several hours, sophisticated probe design, and show relatively low sensitivity for the detection of low-abundance methylated DNA.

It is worth nothing that compared with conventional isothermal amplification protocols such as RCA and SDA, the isothermal exponential amplification reaction (EXPAR) shares several distinct advantages of rapidness, simple design and ultrahigh sensitivity. EXPAR can achieve 10^6^ to 10^9^-fold exponential amplification of target nucleic acids within minutes^33^ by using only one simple DNA template. Fascinatingly, we notice that the GlaI-cleaved methylated DNA end fragments are well suited to serve as triggers to efficiently initiate EXPAR, while the intact unmethylated DNA after GlaI treatment will not be amplified at all by EXPAR. Therefore, by integrating the advantages of GlaI for highly specific methylation discrimination and EXPAR for highly efficient signal amplification, in this work, we have developed a novel GlaI-assisted EXPAR assay (GlaI–EXPAR) that allows the direct quantification of methylated DNA with ultrahigh sensitivity and accuracy. By real-time monitoring of the fluorescence signal of the GlaI–EXPAR system, only the methylated DNA is quantitatively and faithfully reflected while the unmethylated DNA cannot trigger EXPAR and thus will not interfere with the methylation analysis. With the elegant GlaI–EXPAR assay, as low as 200 aM methylated DNA can be clearly detected and the whole detection process can be accomplished within a short time of 2 h. More importantly, due to the excellent selectivity of GlaI towards the methylation site, ultrahigh specificity is also achieved and as low as 0.01% methylated DNA can be clearly identified in the presence of a vast excess of unmethylated DNA. Therefore, the proposed GlaI–EXPAR approach provides a powerful and reliable tool for the rapid, sensitive, highly specific, and accurate detection of site-specific DNA methylation especially for those with extremely low abundances.

## Experimental section

### Reagents and apparatus

GlaI endonuclease as well as its reaction buffer was supplied by SibEnzyme Ltd (Russia). Nt.BstNBI nicking endonuclease (10 000 U mL^–1^), Vent (exo^–^) DNA polymerase (2000 U mL^–1^), M.SssI CpG methyltransferase (4000 U mL^–1^), and *S*-adenosylmethionine (SAM, 32 mM) were all purchased from New England Biolabs (NEB, USA). TKS Gflex DNA polymerase (1250 U mL^–1^), Gflex PCR Buffer, dNTPs (2.5 mM) and the 100 bp DNA ladder marker were obtained from TaKaRa Biotechnology (Dalian, China). 4S Red Plus Nucleic Acid Stain (Life Science, USA) was used for DNA staining in agarose gel electrophoresis. SYBR Green I (20× stock solution in DMSO, 20 mg mL^–1^) was purchased from Bio-Vision Biotechnology (Xiamen, China). All of the oligonucleotides (see detailed sequences in Table S1†) used in this work were custom synthesized by TaKaRa Biotechnology (Dalian, China).

### Extraction of genomic DNA from HCT116 cells

HCT116 cells were obtained from the cell bank of the Chinese Academy of Sciences (Shanghai, China). The cells were cultured in the DMEM medium mixed with 10% fetal calf serum, 100 U mL^–1^ penicillin, 1% NaHCO_3_, 3 mM l-glutamine and 100 mg mL^–1^ streptomycin at 37 °C with appropriate humidified atmosphere (5% CO_2_ and 95% air). After trypsinization (0.2% trypsin, 1 mM EDTA, Invitrogen), about 5 × 10^6^ adherent HCT116 cells, which were counted and quantified by a hemocytometer (Shanghai Qiujing, China), were centrifuged at 11 200 g for 1 min at room temperature. Then the supernatant was carefully discarded and the cell precipitates were collected to be used for the extraction of genomic DNA. Finally, 120 μL of genomic DNA extracts were obtained by using the TIANamp genomic DNA kit according to the manufacturer’s instructions.

### Preparation of unmethylated DNA target (target N) and methylated DNA target (target M)

In order to make the test results more convincing, a 508 bp DNA fragment in septin 9 gene (see detailed sequence in Table S1†) was used as a proof-of-concept target in this study, which was obtained by amplifying the septin 9 gene in the extracted genomic DNA with PCR. Typically, in a 50 μL PCR solution (1× Gflex PCR buffer containing 1 mM Mg^2+^), an appropriate amount of the genomic DNA extracts was mixed with 0.2 mM dNTPs, 1.25 U TKS Gflex DNA polymerase, and 0.2 μM each of the primers (forward primer and reverse primer, see detailed sequences in Table S1†), and then the PCR amplification was performed with the following procedures: 94 °C for 1 min, followed by 30 cycles of 98 °C for 10 s, 60 °C for 15 s, and 68 °C for 30 s. A 2720 thermal cycler (Applied Biosystems, USA) was used for the PCR amplification and the PCR products were characterized by agarose electrophoresis analysis (Fig. S1 in ESI†).

The 508 bp septin 9 gene promoter sequences in the PCR products were the desired target DNA in this study. After agarose electrophoresis, the desired target DNA could be separated and purified from the PCR products by using a QIAquick® Gel Extraction Kit. The purified target DNA was quantified and divided into two equal parts. One part of the target DNA was directly used as the unmethylated DNA target (target N) without any treatment. Meanwhile, the other equivalent part was treated by the M.SssI CpG methyltransferase to produce the methylated DNA target (target M) because the CpG dinucleotide sites on the target DNA were methylated under the catalysis of M.SssI methyltransferase. Typically, a total 50 μL reaction media of 1× NEB buffer 2 (10 mM Tris–HCl containing 50 mM NaCl, 10 mM MgCl_2_ and 1 mM DTT, pH 7.9), 16 U of M.SssI methyltransferase and 32 nmol (equal to a concentration of 640 μM) of SAM were incubated with 2 μM target DNA at 37 °C for 5 h. Afterward, to ensure that all of the target DNA was completely methylated at the CpG dinucleotide sites, 16 U of M.SssI methyltransferase and 32 nmol (640 μM) of SAM were refilled in the mixture and incubated at 37 °C for another 12 h, followed by inactivation of the M.SssI methyltransferase at 65 °C for 20 min. A MinElute® PCR Purification Kit was used for the purification of the methylated DNA according to the manufacturer’s instruction, and the final obtained target M was quantified on a NANODROP 2000 instrument.

### Standard GlaI–EXPAR protocols

In a typical GlaI–EXPAR reaction, series dilutions of target DNAs (target M, target N, or 1 μL of 1000-fold diluted genomic DNA extracts) were firstly incubated with 1 U of GlaI endonuclease at 30 °C for 45 min in a 5 μL reaction buffer (33 mM Tris-HAc, 10 mM Mg(Ac)_2_, 66 mM KAc and 1 mM DTT, pH 7.9). After heating at 70 °C for 15 min to deactivate GlaI, 1 μL of the GlaI-treated mixture was pipetted out and further mixed with the EXPAR template and dNTPs in the Nt.Bst NBI buffer. The mixture was heated at 94 °C for 1 min and then incubated at 55 °C for 5 min. Afterward, Vent (exo^–^) DNA polymerase, Nt.BstNBI nicking endonuclease, SYBR Green I and ThermoPol reaction buffer was further introduced to make a final 10 μL volume for EXPAR. The final 10 μL mixture containing 1× ThermoPol reaction buffer (20 mM Tris–HCl, 10 mM KCl, 10 mM (NH_4_)_2_SO_4_, 2 mM MgSO_4_ and 0.1% Triton X-100, pH 8.8), 0.5× Nt.Bst NBI buffer (25 mM Tris–HCl, pH 7.9, 5 mM MgCl_2_, 50 mM NaCl, 0.5 mM dithiothreitol), 200 μM dNTPs, 0.01 μM EXPAR template, 0.4× SYBR Green I, Vent (exo^–^) DNA polymerase (0.2 U), and Nt.BstNBI nicking endonuclease (4 U) was immediately placed in a Step-One Real-Time PCR system (Applied Biosystems, USA) to perform the EXPAR at 60 °C. The real-time fluorescence intensity was monitored at intervals of 30 s for the quantitative determination of the methylated target DNA.

## Results and discussion

### Principle of the GlaI–EXPAR method for the detection of DNA methylation

**Fig. 1:**
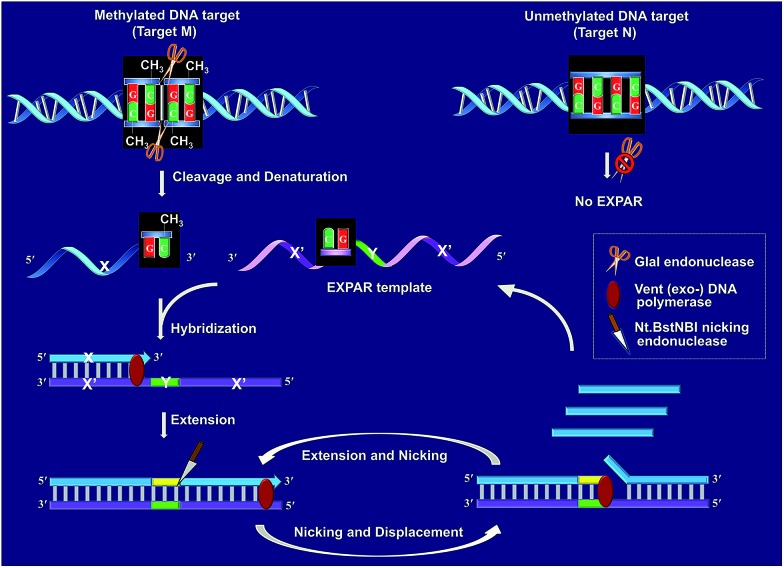
Schematic illustration of the GlaI–EXPAR method for the detection of site-specific DNA methylation.

Fig. 1 schematically illustrates the work principle of the GlaI–EXPAR approach for the detection of site-specific DNA methylation, which takes advantage of the high specificity of GlaI for the digestion of methylated cytosine sites and the high sensitivity of EXPAR for efficient amplification and detection of the GlaI-cleaved products. In this study, a specific DNA sequence in the septin 9 gene containing a 5′-GmCGmC-3′/3′-mCGmCG-5′ site (77373474 site) with four 5-methylcytosines (mC), is employed as a model site-specific methylated DNA target because this methylation site is well-recognized to be closely associated with the tumorigenesis of human colon cancer.^24^ As shown in Fig. 1, two simple steps, the GlaI-based digestion of methylated DNA and sequential EXPAR amplification, are involved in the GlaI–EXPAR assay. First, when the methylated DNA (target M) or the unmethylated DNA (target N) were incubated with the GlaI, the endonuclease can specifically recognize the methylated DNA site 5′-GmCGmC-3′/3′-mCGmCG-5′ and cut it in the middle of the sequence GmCGmC. Meanwhile, the unmethylated 5′-GCGC-3′/3′-CGCG-5′ site in target N cannot be recognized by GlaI and thus target N will remain intact.

After GlaI treatment and subsequent heating denaturation, the newly exposed 3′-end sequence (X) of the digested target M is able to initiate EXPAR for quantitative methylation analysis. The EXPAR template (X′–Y–X′) is specifically designed to consist of two repeat X′ sequences (complementary to X) separated by an Nt.BstNBI nicking endonuclease recognizing sequence Y in the middle. In the GlaI–EXPAR system, the EXPAR template is present at a rather high concentration, which is largely excess compared with that of the detected DNA target. So the newly exposed 3′-end X sequence has a predominantly higher chance to hybridize with the EXPAR template rather than with its original complementary X′ fragment. So the high concentration of EXPAR template is important and necessary in this study for the accurate quantification of site-specific DNA methylation. In this regard, the X sequence at the 3′-end of the digested target M will hybridize with X′ at the 3′-terminus of the EXPAR template and then extend along the EXPAR template to form an elongated double-stranded DNA (dsDNA) under the catalysis of Vent (exo^–^) DNA polymerase. Afterward, Nt.BstNBI nicking enzyme will specifically recognize the nicking site and cleave the upper strand of the newly formed dsDNA. The cleaved strand containing the recognition site will extend again along the EXPAR template to release a new X sequence owing to the strand-displacement activity of Vent (exo^–^) DNA polymerase. Then, the extension, nicking, and strand-displacement will repeat to generate a lot of X sequences. Meanwhile, the newly released X can also hybridize with other free EXPAR templates to initiate new cycles of extension, nicking and strand-displacement reactions, leading to rapid exponential signal amplification. Through real-time fluorescence detection of the EXPAR products by using SYBR Green I, the site-specific methylated target DNA can be quantitatively and sensitively determined.

In contrast, since GlaI is highly methylation-dependent, the unmethylated target N will keep intact after treatment with GlaI. As a result, the X sequence in the target N will not be exposed to the 3′-end and thus no EXPAR amplification will be initiated. Therefore, unlike the traditional MSRE-based methylation assays where false positive interferences are inevitable, in the proposed GlaI–EXPAR strategy, the potential interference from the unmethylated DNA is effectively avoided so that the site-specific DNA methylation can be more accurately and faithfully detected.

### Feasibility evaluation of the GlaI–EXPAR method for the detection of methylated DNA

**Fig. 2:**
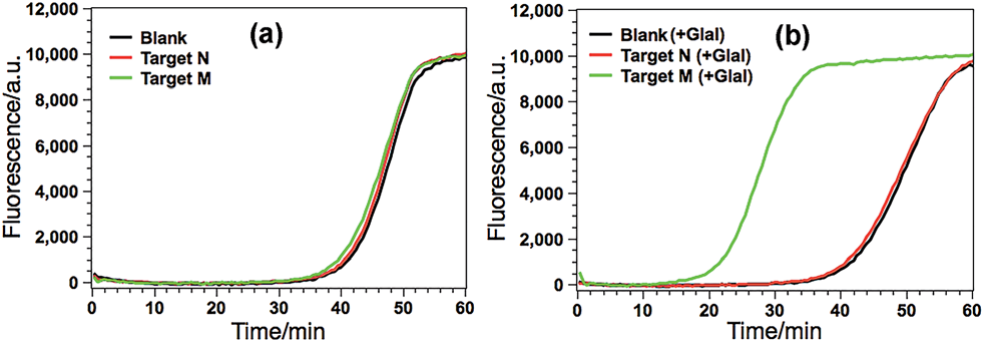
(a) Real-time fluorescence curves of the GlaI–EXPAR produced by blank (black line), 2 pM target N (red line) and 2 pM target M (green line) without GlaI treatment; (b) real-time fluorescence curves of the GlaI–EXPAR produced by blank (black line), 2 pM target N (red line) and 2 pM target M (green line), which are all treated with 1 U of GlaI. Other experimental conditions are the same as those described in the Experimental section. The blank is detected by the same procedures but without adding any DNA target.

According to the design principle illustrated in Fig. 1, the ability of GlaI for the highly specific methylation discrimination is most crucial for the proposed GlaI–EXPAR assay. So verification experiments were first conducted to evaluate the critical role of GlaI and the feasibility of the proposed GlaI–EXPAR methylation assay. As displayed in Fig. 2a, without the GlaI treatment, both target M and target N cannot trigger EXPAR and the real-time fluorescence signals produced by 2 pM target M and target N are almost the same as that of the blank control. In contrast, with the methylation-selective GlaI digestion, as shown in Fig. 2b, target M produces a significant positive response due to the efficient EXPAR amplification while the fluorescence curve aroused by target N is still overlapped with the blank control, indicating that GlaI-treated target N still cannot initiate any EXPAR amplification. These results clearly verify the excellent ability of the GlaI for the highly selective digestion of methylated DNA against the unmethylated ones. To further support the feasibility of the GlaI–EXPAR methylation assay, the amplification products of the GlaI–EXPAR system produced by target M, target N or blank control are all characterized by polyacrylamide gel electrophoresis (PAGE, Fig. S2†). The PAGE results are well consistent with the real-time fluorescence results. All of these results clearly indicate that the GlaI–EXPAR method is undoubtedly feasible for the accurate and highly specific detection of site-specific DNA methylation.

### Analytical performance of the GlaI–EXPAR method for target M detection

Various experimental parameters including the amount of the involved enzymes and the temperature of the EXPAR, which may influence the performance of the GlaI–EXPAR assay for the detection of target M, were all investigated and optimized in detail (Fig. S3–S6 in ESI†). Accordingly, 1 U GlaI endonuclease, 4 U Nt.BstNBI nicking enzyme, 0.2 U Vent (exo^–^) DNA polymerase, and the EXPAR temperature of 60 °C are found to be the optimal conditions.

**Fig. 3:**
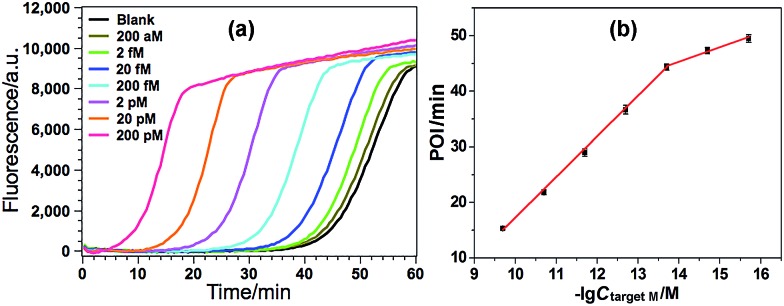
(a) The real-time fluorescence curves produced by target M with different concentrations. From left to right, the target M concentrations are successively 200 pM, 20 pM, 2 pM, 200 fM, 20 fM, 2 fM, 200 aM and 0 (blank); (b) the plots between POI values in the real-time fluorescence curves and logarithm (lg) of the target M concentrations. Error bars are estimated from the standard deviation of three replicate measurements at each data point.

Under such optimized experimental conditions, the analytical performance of the proposed GlaI–EXPAR method for the detection of target M was evaluated. Fig. 3a exhibits the well-defined real-time fluorescence curves produced by different concentrations of target M ranging from 200 aM to 200 pM, respectively. It can be seen that with increasing concentrations of target M, the fluorescence curves arise more rapidly since the detected EXPAR products are only dependent on the initial dosage of target M. The point of inflection (POI) values, namely, the times corresponding to the maximum slope in each real-time fluorescence curve, are recorded for the quantitative determination of target M. As shown in Fig. 3b, two good linear relationships are obtained between the POI values and logarithm of the target M concentrations in the ranges of 200 aM to 20 fM, and 20 fM to 200 pM, respectively. The corresponding correlation equations are POI = 8.68 – 2.61 lg(*C*_target M_/M) (correlation coefficient *R* = 0.9811) and POI = –54.41 – 7.18 lg(*C*_target M_/M) (*R* = 0.9986), respectively. The results shown in Fig. 3a clearly demonstrate that the fluorescence response produced by as low as 200 aM target M (equal to an absolute quantity of 2 zmol methylated DNA molecules in a 10 μL volume) can be clearly discriminated from the blank, indicating an ultrahigh sensitivity of the proposed GlaI–EXPAR assay. Furthermore, compared with the most widely used BC-based methylation assays which typically need 16–40 h assaying time^34^ due to their cumbersome procedures, the whole GlaI–EXPAR assay can be accomplished within 2 h including both the simple GlaI treatment and EXPAR reaction.

**Fig. 4:**
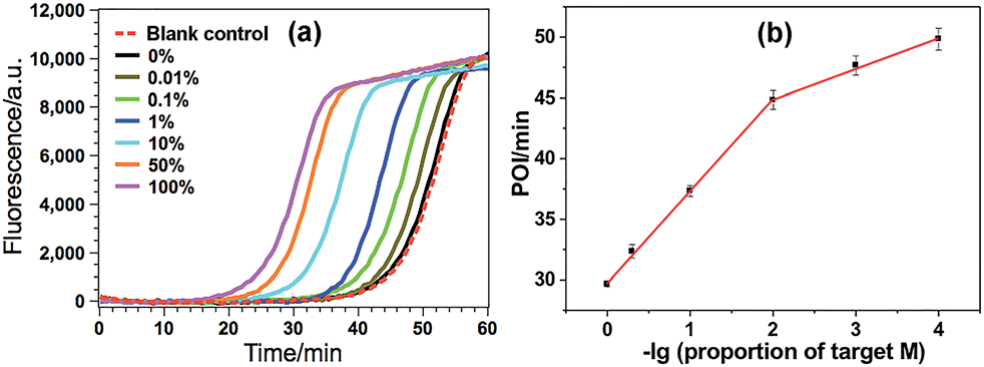
(a) The real-time fluorescence curves produced by the mixture of target M and target N (total concentration of 2 pM) with varying ratios. From left to right (solid lines), the ratio of target M in the mixtures is 100, 50, 10, 1, 0.1, 0.01 and 0%, respectively. Meanwhile, the fluorescence curve produced by the non-target blank control, namely, the blank control without adding either target M or target N, is also recorded for comparison (red dashed line). The experimental conditions are the same as those in Fig. 3; (b) the relationship between the POI values in the real-time fluorescence curves and logarithm (lg) of the ratio of target M in the total DNA. Error bars are estimated from the standard deviation of three replicate measurements.

The high specificity to discriminate low levels of methylated target in the presence of a high background of matched normal DNA is extremely important for a practical methylation assay. In this study, target M is mixed with target N at different ratios with a constant total DNA concentration, in which the ratio of target M in the total DNAs varies from 0 to 100%. Such mixtures are directly used as the samples to test the specificity of the proposed GlaI–EXPAR methylation assay. As can be seen from Fig. 4a that the real-time fluorescence curve produced by the DNA sample containing only target N (0% of target M) is almost the same as that aroused by the non-target blank control, namely, the blank control without adding either target M or target N. Meanwhile, one can see that with higher ratio of target M in the DNA mixture, the fluorescence curve arises more rapidly. Such results suggest that the positive fluorescence responses observed in Fig. 4a are indeed only aroused by target M in the DNA mixture, clearly proving the high specificity of the GlaI–EXPAR assay for the detection of target M. Furthermore, the POI values of the real-time fluorescence curves are linearly proportional to the ratios of target M in the ranges of 0.01–1%, and 1–100%, respectively (Fig. 4b). Accordingly, the correlation equations are POI = 39.91 – 2.52 lg(proportion of target M) (*R* = 0.9873), and POI = 29.58 – 7.55 lg(proportion of target M) (*R* = 0.9957), respectively. Such standard calibration curves may be applied to the direct evaluation of site-specific methylation ratios in mixed DNA samples with a known total concentration. Notably, one can see from Fig. 4 that as low as 0.01% target M (equal to a rather low concentration of 200 aM) can be clearly detected by the GlaI–EXPAR method in the presence of a large excess of unmethylated target N, suggesting an ultrahigh specificity for the selective detection of methylated DNA target in a large amount of DNA pool. It is worth noting that for most of the conventional BC- or restriction endonuclease-based methylation assays, such as eMethylsorb,^35^ MALDI-MS spectrometry,^36^ HRMA method,^37^ cationic conjugated polymer-based method,^38^ SMART-MSP^22^ and HDCR,^24^ only 0.1% to 10% methylated target DNA can be detected in the DNA mixtures containing excessive unmethylated DNA. So the specificity of the proposed GlaI–EXPAR is superior to these traditional methylation assays. As far as we know, only seldom reported methods such as the MS-qFRET^39^ and HRCA^29^ are capable of discerning 0.01% methylated target DNA from their unmethylated counterparts, showing comparable specificity to the GlaI–EXPAR method. However, compared with the GlaI–EXPAR method, both MS-qFRET and HRCA need stringent primer/probe design, complicated procedures, long reaction time and show relatively low sensitivity. Therefore, due to the excellent specificity, high sensitivity and simple operations, the proposed GlaI–EXPAR assay provides a powerful and reliable protocol for the detection of site-specific DNA methylations.

### Detection of DNA methylation in real genomic DNA samples

**Fig. 5:**
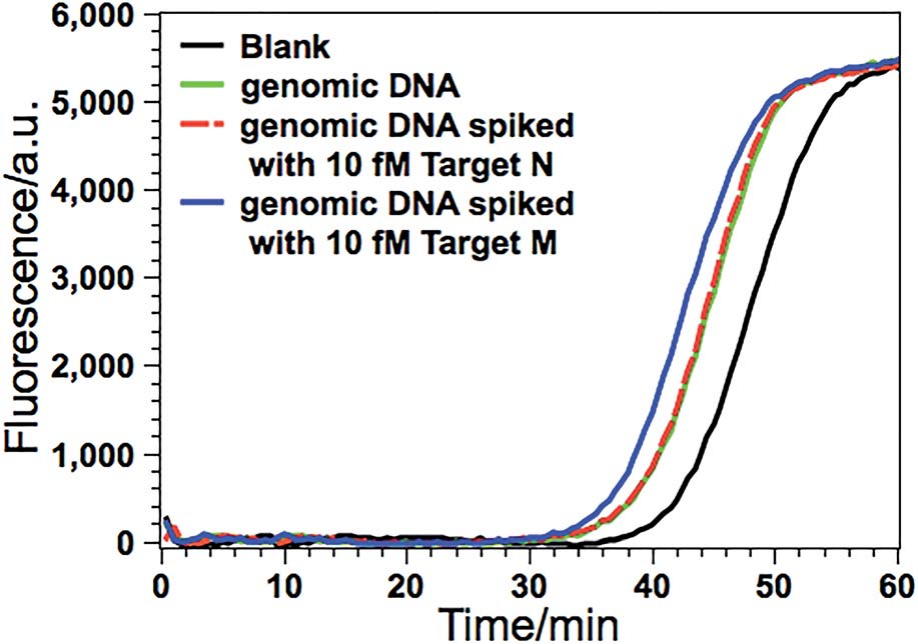
Detection of target M (77373474 site in septin 9 gene) in the genomic DNA sample extracted from HCT116 cells. The real-time fluorescence curves are produced by the genomic DNA sample (green line), the genomic DNA sample spiked with 10 fM standard target M (blue line), the genomic DNA sample spiked with 10 fM target N (red dashed line), and the blank control (black line), respectively. The determined concentration of target M in genomic DNA is calculated in the 10 μL GlaI–EXPAR system. Error bars are estimated from three replicate measurements.

Due to its ultrahigh sensitivity and specificity, the GlaI–EXPAR method is further examined for the detection of site-specific methylation in real genomic DNA samples extracted from the HCT116 colon cancer cells. The level of the methylated DNA target in 1 μL of 1000-fold diluted genomic DNA extracts is quantitatively evaluated according to the calibration curve as that shown in Fig. 3b. As displayed in Fig. 5, the POI value produced by the genomic DNA sample falls within the linear range of 200 aM to 20 fM, and thus the amount of target M in the genomic DNA sample is determined to be 3.1 fM according to the corresponding calibration equation. When the genomic DNA sample is further spiked with 10 fM of standard target M, the time of the POI value is remarkably shortened, and the amount of target M in the spiked sample is calculated to be 12.4 fM with a recovery of 93.0%. In contrast, when the equivalent genomic DNA sample is spiked with 10 fM of unmethylated target N, the obtained POI value is exactly identical to that produced by only the genomic DNA sample (red dashed line in Fig. 5). These results clearly suggest that the positive responses of the genomic DNA samples are indeed faithfully aroused by the site-specific methylated target sequence. So this GlaI–EXPAR assay is feasible and reliable for the highly sensitive and highly specific detection of minute amounts of site-specific DNA methylations in real genomic DNA samples. To further prove the accuracy of the obtained results, the detected 5′-GCGC-3′/3′-CGCG-5′ site (77373474 site) of the target septin 9 gene sequence in the extracted genomic DNA from HCT-116 cells is sequenced (Fig. S7, ESI†). The bisulfite sequencing result indicates that the cytosines in this site are all methylated. Therefore, the detection results obtained by the GlaI–EXPAR are consistent with the sequencing results.

### Evaluation of the generality of the GlaI–EXPAR method for methylation analysis

According to a previous study by the Tarasova group,^19^ three types of methylated sequence sites can serve as good substrates of GlaI. The list of favorable cleavage sites of GlaI includes a fully methylated site 5′-GmCGmC-3′/3′-mCGmCG-5′, a general structure 5′-PumCGmC-3′/3′-PyGmCG-5′ with three methylated cytosines (Pu stands for purine nucleotides while Py stands for pyrimidine nucleotides), and one recognition sequence with two methylated cytosines 5′-AmCGT-3′/3′-TGmCA-5′, all of which include at least one methylated CpG dinucleotide. So by combining with the cleavage site-specific EXPAR template, the proposed GlaI–EXPAR can be applicable for the quantitative detection of GlaI-recognizing site-specific CpG methylation.

**Fig. 6:**
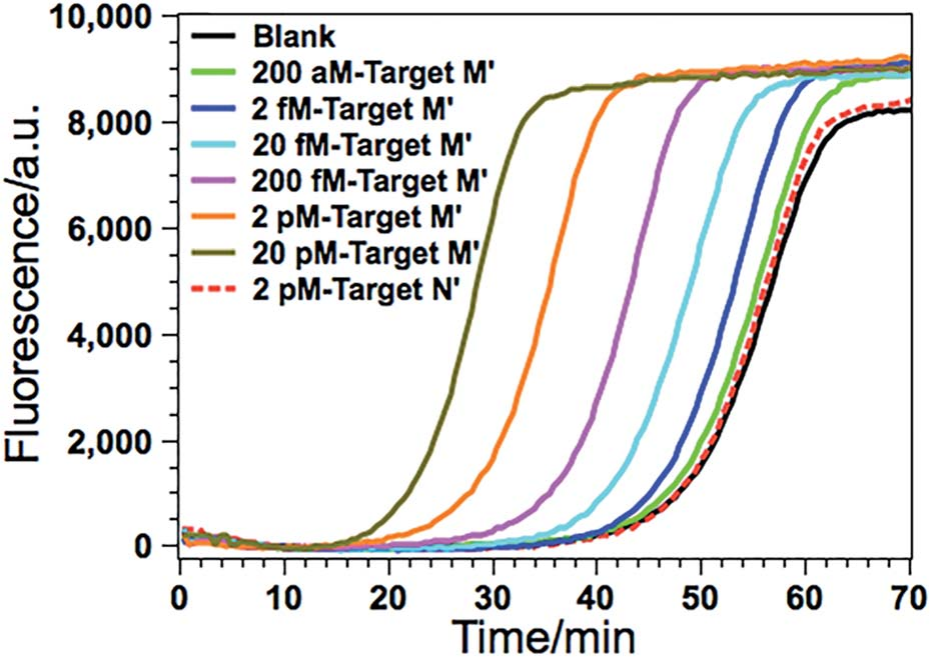
The real-time fluorescence curves for the detection of the 77373518-site methylation in the target DNA (target M′). From left to right, the concentrations of the target M′ are successively 20 pM, 2 pM, 200 fM, 20 fM, 2 fM, 200 aM and 0 (blank), respectively. Meanwhile, the fluorescence curve produced by 2 pM unmethylated target N′ is also recorded for comparison (red dashed line). The experimental conditions are all the same as those in Fig. 3 except for the methylation site-specific EXPAR template.

The detected proof-of-concept target M (77373474 site) is a fully methylated 5′-GmCGmC-3′/3′-mCGmCG-5′ site. To test the generality of the proposed method, the GlaI–EXPAR strategy is further applied to the detection of another consensus GlaI-recognizing methylation site (77373518 site, denoted as target M′) with a 5′-AmCGmC-3′/3′-TGmCG-5′ sequence in the hypermethylated region of the septin 9 gene, which is one of the best GlaI substrates in the general 5′-PuCGC-3′/3′-PyGCG-5′ structures.^19^ According to the principle of GlaI–EXPAR, for the detection of different methylation sites, only the “X” sequence of the EXPAR template need to be changed according to the site-specific 3′-end sequence of the GlaI-cutting site, while other experimental conditions can stay the same. In this study, the GlaI-digested target M′ exposes a new end sequence (Z) at the 3′-terminus, which can initiate EXPAR for quantitative methylation analysis by using the site-specific EXPAR template (Z′–Y–Z′, see detailed sequence in Table S1†). As can be seen from the real-time fluorescence curves in Fig. 6, with increasing concentrations of target M′ from 200 aM to 20 pM, the corresponding POI value is gradually shortened, and as low as 200 aM target M′ can be unequivocally detected from the blank control. Fig. S8† shows that the POI values are linearly proportional to the logarithm of the target M′ concentrations in the ranges of 200 aM to 20 fM, and 20 fM to 20 pM, respectively. The corresponding correlation equations are POI = 20.02 – 2.18 lg(*C*_target M′_/M) (*R* = 0.9920) and POI = –56.91 – 7.86 lg(*C*_target M′_/M) (*R* = 0.9956), respectively. Such results are in good consistence with those for the detection of target M (Fig. 3), suggesting that the GlaI–EXPAR can be easily extended to the detection of different site-specific DNA methylations with similar high sensitivity. Notably, one can also see from Fig. 6 that the fluorescence response aroused by unmethylated target N′ with a high concentration of 2 pM is almost the same with the blank control, further verifying the ultrahigh specificity of the GlaI which is capable of accurately discriminating methylated target DNA from a large amount of 10^4^-fold excess of unmethylated DNA.

## Conclusions

In summary, by integrating the distinct advantages of GlaI endonuclease for highly selective recognition and cleavage of methylated DNA target, and EXPAR for the highly efficient amplification of GlaI-cleaved DNA fragments, we have for the first time developed a novel GlaI–EXPAR strategy which allows for the quantitative evaluation of site-specific DNA methylation with ultrahigh sensitivity and specificity. Compared with the conventional MSRE-based methylation assays, since the GlaI can only recognize and cut the methylated DNA site and then only the GlaI-digested DNA fragments can initiate subsequent EXPAR, the combination of GlaI with EXPAR can efficiently avoid the potential false positive interference from unmethylated target DNA or non-target DNAs. Furthermore, different from the current gold-standard BC-based methylation assays where cumbersome and time-consuming operations are generally involved, in the GlaI–EXPAR assay, the operations for both the GlaI treatment and EXPAR are quite simple and rapid. Highly sensitive (with a detection limit down to the aM level) and specific (0.01% methylated target can be discerned from a large DNA pool) methylation detection can be readily accomplished within 2 h under isothermal conditions. Considering the high sensitivity, excellent specificity, easy probe design and simple operation, we believe that this proposed GlaI–EXPAR strategy may provide a robust and reliable platform for the accurate detection of site-specific DNA methylations in both biological and biomedical studies.

## Conflicts of interest

There are no conflicts to declare.

## Supplementary Material

Supplementary information
