# Supplementary material for: A novel restriction endonuclease GlaI for rapid and highly sensitive detection of DNA methylation coupled with isothermal exponential amplification reaction
Source: Chem Sci. 2017 Dec 11;9(5):1344–51. doi: 10.1039/c7sc04975g (PMC5887237; doi:10.1039/c7sc04975g)
Supplement: Supplementary file 1 [file SC-009-C7SC04975G-s001.pdf]

Electronic Supplementary Material (ESI) for Chemical Science.
This journal is © The Royal Society of Chemistry 2017

Electronic Supplementary Information

A novel restriction endonuclease GlaI for rapid and highly sensitive detection of DNA methylation with isothermal exponential amplification reaction

Yueying Sun, Yuanyuan Sun, Weimin Tian, Chenghui Liu,* Kejian Gao, Zhengping Li*

Key laboratory of Applied Surface and Colloid Chemistry, Ministry of Education, Key Laboratory of Analytical Chemistry for Life Science of Shaanxi Province, School of Chemistry and Chemical Engineering, Shaanxi Normal University, Xi'an 710062, Shaanxi Province, P. R. China

Email: liuch@snnu.edu.cn; lzpbd@snnu.edu.cn

List of Contents:

1. The DNA sequences used in this study (Table S1)

2. Agarose electrophoresis results of the PCR products amplified from the extracted genomic DNA

3. Polyacrylamide gel electrophoresis result of the GlaI-EXPAR

4. Effect of the amount of GlaI endonuclease on the detection of DNA methylation

5. Optimization of the amount of Nt.BstNBI nicking endonuclease

6. Optimization of the amount of Vent (exo-) DNA polymerase

7. Optimization of the temperature of the EXPAR

8. Bisulfite sequencing result of the target DNA sequence in Septin 9 gene extracts from HCT116 cells

9. The plots between POI values in the real-time fluorescence curves and logarithm (lg) of the target M' concentrations

1. The DNA sequences used in this study

Table S1. The DNA sequences used in this study.

Name	Sequence (5'-3' direction)
Forward primer	TAACAGGGTCCCCCACGTAG
Reverse primer	GTGCGGGTGCGGGAACCTGA
EXPAR template for target M (77373474 site)	GCGACCCGCTGCCCACCAGCCA*AACAGACTC*GCGACCCGCTGCC<br>**X'** **Y** **X'**<br>CACCAGCCATTTTTTTTTT-SH
EXPAR template for target M' (77373518 site)	GCGCAGCTGGATGGGATCATTTCGGACTTC*AACAGACTC*GCGCA<br>**Z'** **Y**<br>GCTGGATGGGATCATTTCGGACTTCTTTTTTTTTT-SH<br>**Z'**
Target DNA (508 bp)	TAACAGGGTCCCCCACGTAGGCGGCGCGGGCACGGGGCCGGGGG<br>CGCGCGGCGCTCTCAGCGGGAGGGCGCCTGGATTAGGGGTCCCG<br>TCTCCCCTCAGCGCACTCCCGTCTCCCCTCAGCGCACCCCCGTCCC<br>GCGCCAGGCCCACCCGCAGGGTCCTCTCCAGCACGTCCGCGGCCG<br>CAGCAGCCAGCCCAGCACCCACCTTCGAAGTCCGAAATGATCCC<br>ATCCAGCTGC***GCGT***TGACCGCGGGGTCCGACATGATGGCTGGTGG<br>Z<br>GCAGCGGGTC***GCGC***GGAGGGCAGCGGCGAGGAAGCGCCCCCGGC<br>X<br>GGGGCCCGGGCCCTGCGCGCTGGCTGGGGCGCCGCGCCCGCGCT<br>CCTGCAGTGCAGAGCTAGCCGCCGGAGGAGCCCCTAGGCCCCCT<br>GGCTCAGCTGAATGAATGGGGGAGGAAGGCGGGCGCCGGCCCCT<br>CCCCGCGCGCTGCGCCCCCGCCCCGCCCCCGCCTCCCGGGCGGAT<br>CAGGTTCCCGCACCCGCAC

Note: Forward primer and Reverse primer were used for the PCR for preparation of the target DNA from genomic DNA. In target DNA, the GCGC (target M) and GCGT (target M') sites highlighted in red color are the detected methylation sites (the methylated cytosines are also highlighted by green background) in this study, which sites can be recognized by GlaI endonuclease.

2. Agarose electrophoresis results of the PCR products amplified from the extracted genomic DNA

After PCR, agarose electrophoresis analysis was performed to characterize the PCR products, which was carried out in 2% (w/v) agarose gels with 1× TAE buffer (pH 8.5). Typically, the mixtures containing 1 μL of 6× loading buffer and 5 μL of PCR products were prepared and then 5 μL of such

mixtures were loaded on the gel. Electrophoresis was run at a constant voltage 110 V for 50 min, and then the gel was stained by 4S Red Plus Nucleic Acid Stain and visualized on a Gel Doc EZ Imager.

The result shown in Fig. S1 has clearly demonstrated that the target DNA carrying 508-bp of Septin 9 gene was amplified and prepared successfully.

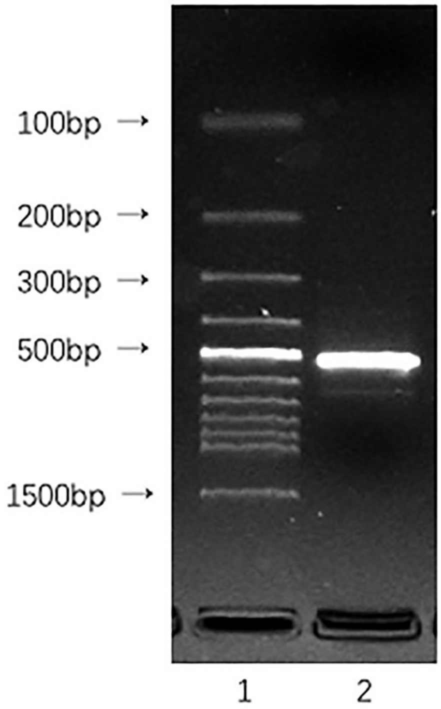

Fig. S1. Agarose electrophoresis results of the PCR products. Lane 1, DNA ladder; Lane 2, the as-prepared target DNA after PCR of the genomic DNA.

3. Polyacrylamide gel electrophoresis result of the GlaI-EXPAR

To further support the feasibility of the GlaI-EXPAR assay for the detection of target M, the amplification products of the GlaI-EXPAR system produced by target M, target N or blank control (with a reaction time of 35 min) were all characterized by polyacrylamide gel electrophoresis (PAGE, Fig. S2). As shown in Fig. S2, both the pure EXPAR template (lane 3) and the pure single-stranded X sequence (lane 2) show their defined bands, respectively. In the GlaI-EXPAR system, only the distinct band corresponding to pure EXPAR template can be observed for both the blank control (lane 4) and the samples in the presence of different concentrations of target N (20 pM and 100 pM for lane 5 and

8, respectively), indicating that both the blank control and target N do not trigger EXPAR at all. In contrast, when 20 pM or 100 pM target M is presented, as displayed in lane 6 and lane 7, the band corresponding to the EXPAR template decreases gradually and a new band with a lower mobility than the EXPAR template arises and may become the dominant product at higher concentrations of target M. According to the working principle of GlaI-EXPAR, the new band can be ascribed to the EXPAR template-tethered completely or partially hybridized duplexes. These results are well consistent with the prediction of GlaI-EXPAR products, further indicating the reliability and high specificity of the GlaI-EXPAR assay for the detection of target M.

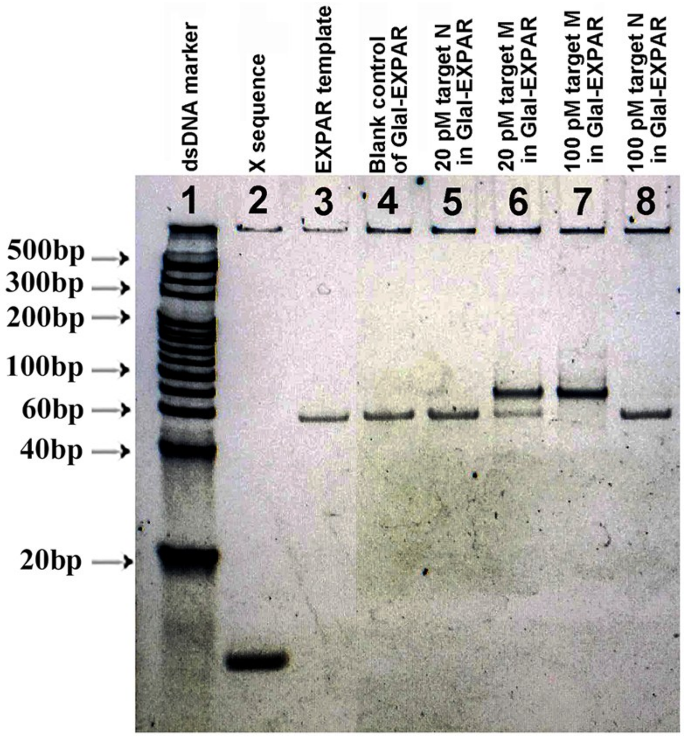

Fig. S2. Non-denaturing PAGE analysis of the amplification products of GlaI-EXPAR reaction. **Lane 1**, double-strand (ds) DNA markers (20 bp DNA ladder); **lane 2**, pure synthetic X sequence (50 nM); **lane 3**, pure EXPAR template (50 nM); **lane 4**, amplification products of GlaI-EXPAR by the blank control (without either target M or target N); **lane 5**, amplification products of GlaI-EXPAR in the presence of 20 pM target N; **lane 6**, amplification products of GlaI-EXPAR in the presence of 20 pM target M; **lane 7**, amplification products of GlaI-EXPAR in the presence of 100 pM target M; **lane 8**, amplification products of GlaI-EXPAR in the presence of 100 pM target N. 50 nM EXPAR template

was used in the GlaI-EXPAR. The gel was stained by 4S Red Plus Nucleic Acid Stain and visualized on a Gel Doc EZ Imager (Bio-Rad, USA).

4. Effect of the amount of GlaI endonuclease on the detection of DNA methylation

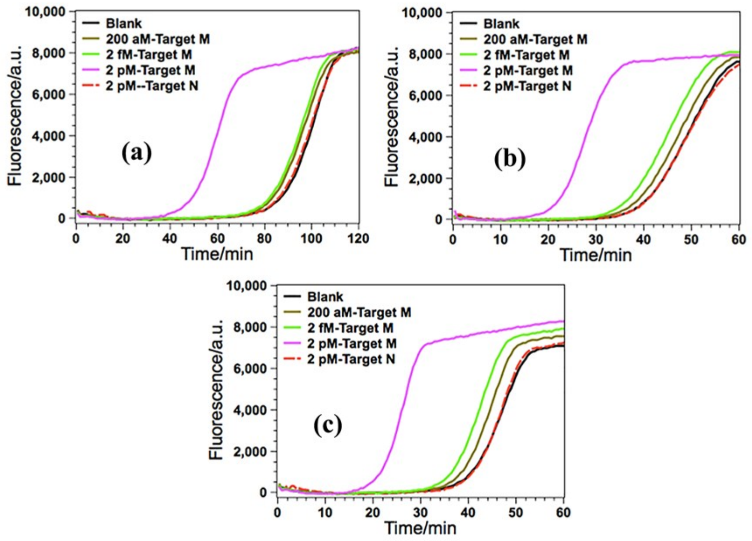

Fig. S3. The effect of the amount of GlaI on the detection of DNA methylation. The real-time fluorescence curves are produced by 0 (blank control), 200 aM, 2 fM, 2 pM target M and 2 pM target N (red dashed line), respectively, in each image. The amount of GlaI endonuclease is (a) 0.5 U, (b) 1 U, and (c) 2 U, respectively. Other experimental conditions are the same as described in the experiment section.

The amount of GlaI endonuclease is a crucial factor for achieving high sensitivity and selectivity for methylation analysis. To test the effect of the amount of GlaI on the detection of DNA methylation, the blank, 200 aM, 2 fM, 2 pM target M and 2 pM target N are simultaneously detected with the GlaI-EXPAR assay by using different amounts of GlaI. As depicted in Fig. S3, all of the POI values produced by different concentrations of target M display the similar target dosage-responsive decrease tendencies with the treatment of 0.5 U, 1 U or 2 U GlaI. Nevertheless, one can see that the differences of POI values between the blank, 200 aM target M and 2 fM target M are much smaller with 0.5 U GlaI compared with those with 1 U or 2 U GlaI. Meanwhile, with the higher GlaI amount of 1 U or 2

U, the real-time fluorescence curves produced by 2 pM target N still remain the same with the blank control, indicating that the high specificity of the GlaI-EXPAR will not be influenced by higher concentrations of GlaI. Therefore, taking into considerations of high sensitivity, assay cost and high specificity, 1 U of GlaI is selected for the GlaI-EXPAR assay.

5. Optimization of the amount of Nt.BstNBI nicking endonuclease

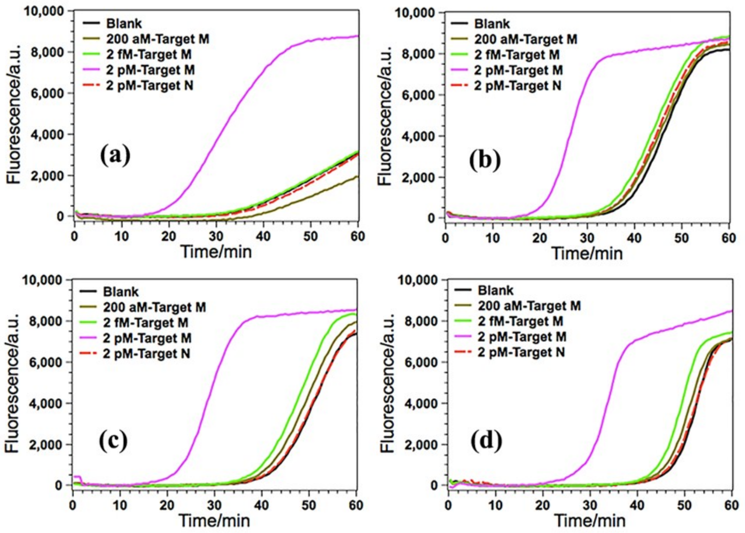

Fig. S4. The influence of the amount of Nt.BstNBI nicking enzyme on the detection of methylated DNA. The real-time fluorescence curves of the GlaI-EXPAR are produced by 0 (blank control), 200 aM, 2 fM, 2 pM target M and 2 pM target N (red dashed line), respectively. The amount of Nt.BstNBI endonuclease used in the GlaI-EXPAR is (a) 2 U, (b) 3 U, (c) 4 U, and (d) 5 U. Other experimental conditions are the same as described in the experiment section.

According to the design principle, the GlaI-EXPAR methylation assay can be also influenced by the amount of Nt.BstNBI nicking endonuclease. So the effect of the amount of Nt.BstNBI nicking enzyme on the performance of the proposed methylation assay is also investigated. As shown in Fig. S4, when 2 U of Nt.BstNBI nicking enzyme is used, the reaction rate of EXPAR is too slow to get well-defined exponential fluorescent curves. When the amount of nicking enzyme increases to 3 U, well-defined exponential fluorescence curves are obtained but the fluorescence response of 200 aM target M cannot be discriminated from the blank control. In contrast, when the Nt.BstNBI enzyme is further elevated to

4 U or 5 U, 200 aM target M can be clearly detected from the blank control and the real-time fluorescence curves produced by 2 pM target N still remain the same with the blank control with maintained ultrahigh specificity. Therefore, taking into considerations of high sensitivity, assay cost and high specificity, 4 U of Nt.BstNBI nicking endonuclease is selected for the GlaI-EXPAR assay.

6. Optimization of the amount of Vent (exo$^-$) DNA polymerase

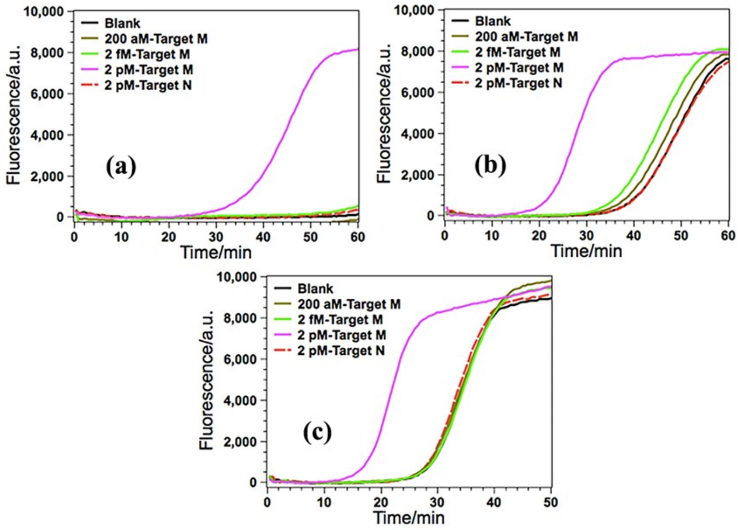

Fig. S5. The influence of the amount of Vent (exo-) DNA polymerase on the proposed methylation assay. The real-time fluorescence curves of the EXPAR-based method are produced by 0 (blank control), 200 aM, 2 fM, 2 pM target M and 2 pM target N (red dashed line), respectively. The amount of Vent (exo-) DNA polymerase used in the EXPAR-based reaction is (a) 0.1 U, (b) 0.2 U, and (c) 0.3 U. Other experimental conditions are the same as described in the experiment section.

In this study, the EXPAR reaction relies on the templated DNA extension and strand displacement catalyzed by Vent (exo-) DNA polymerase with displacement activity. So the amount of DNA polymerase is optimized for the GlaI-EXPAR assay. As shown in Fig. S5, the EXPAR will be gradually accelerated with the increase of the amount of Vent (exo-) DNA polymerase from 0.1 U to 0.3 U. The lower concentration of polymerase will lead to the lower efficiency of EXPAR. As can be seen from Fig. S5a, in the presence of 0.1 U Vent (exo-) DNA polymerase, the rate of EXPAR is quite slow so that the exponential fluorescence curves cannot be well acquired within the reaction time of

60 min, which is not practical for methylation analysis. When the amount of Vent (exo-) DNA polymerase increases to 0.2 U, as displayed in Fig. S5b, well-defined exponential fluorescence curves can be obtained within 60 min and 200 aM target M can be clearly detected. Meanwhile, target N with a high concentration of 2 pM will not interfere with the detection of target M. However, one can see from Fig. S5c that the employment of 0.3 U Vent (exo-) DNA polymerase will lead to the rapid acceleration of nonspecific signal from the blank control, making the low concentrations of target M (200 aM and 2 fM) unable to be detected. In this regard, the amount of 0.2 U Vent (exo-) DNA polymerase is chosen as the optimal amount for the GlaI-EXPAR assay.

7. Optimization of the temperature of the EXPAR

The influence of EXPAR temperature on the proposed methylation assay is further studied and optimized. According to the product manuals from the supplier, the optimum reaction temperatures of Vent (exo-) DNA polymerase and Nt.BstNBI nicking enzyme are 75 °C and 55 °C, respectively. So for the GlaI-EXPAR method, the optimal reaction temperature should fall within the range of 55 °C~75 °C to make sure that both of the two enzymes will keep appropriate activities. So in this study, the EXPAR temperatures of 57 °C, 60 °C and 65 °C are investigated for the methylation analysis. As demonstrated in Fig. S6, when the EXPAR is performed at 57 °C, the fluorescence signals arise rather slowly due to the low efficiency of EXPAR, which may be attributed to the low activity of Vent (exo-) DNA polymerase at 57 °C. While with a high temperature of 65 °C, no detectable exponential amplification signals can be observed at any concentration of target M probably owing to the inactivation of Nt.BstNBI nicking enzyme at such a high temperature (Fig. S6c). Fortunately, when the EXPAR is conducted at 60 °C (Fig. S6b), both of Vent (exo-) DNA polymerase and Nt.BstNBI nicking enzyme will maintain suitable activity so that well-defined exponential fluorescence curves can be obtained, and 200 aM target M can be clearly discriminated from the blank control without the interference of target N. Therefore, 60 °C is selected as the optimal temperature for EXPAR in this study.

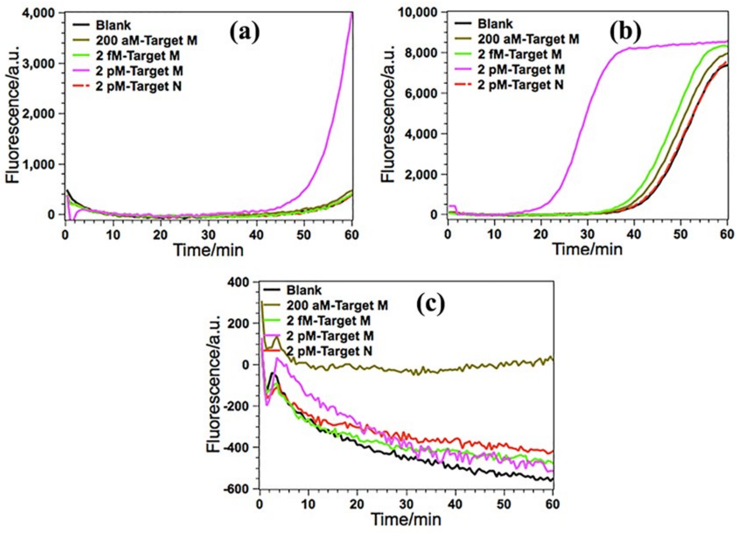

Fig. S6. The effect of EXPAR temperature on the GlaI-EXPAR assay. The real-time fluorescence curves are respectively produced by 0 (blank control), 200 aM, 2 fM, 2 pM target M and 2 pM target N in each image. The reaction temperature is (a) 57 °C, (b) 60 °C, and (c) 65 °C, respectively. Other experiment conditions are the same as described in the experiment section.

8. Bisulfite sequencing result of the target DNA sequence in Septin 9 gene extracts from HCT116 cells

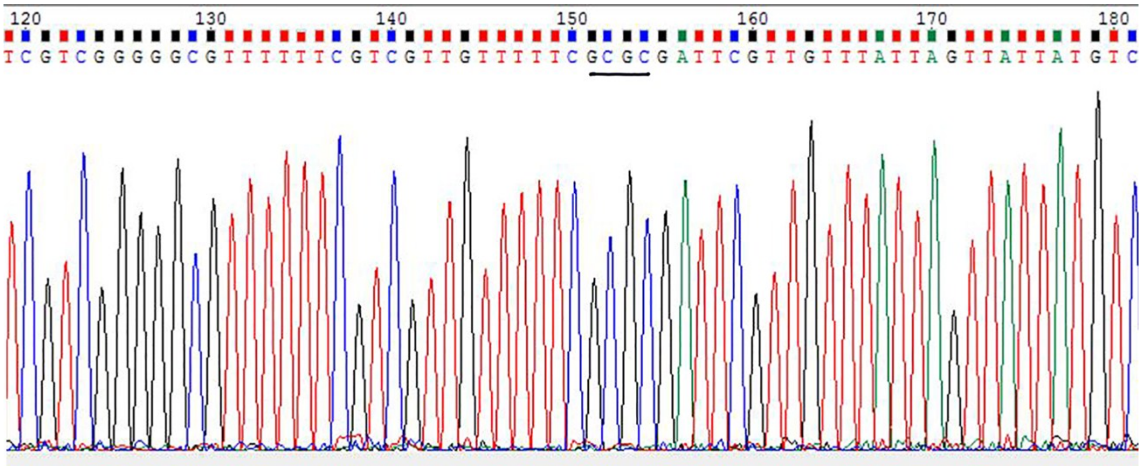

Fig. S7. The bisulfite sequencing result of the target DNA region of Septin 9 gene in which the sequence of the detected methylation site is underlined.

Genomic DNA was extracted from HCT116 cells in our lab by using TIANamp Genomic DNA Kit according to the manufacture's protocol. The extracted genomic DNA was sent to Sangon Biotech. (Shanghai, China) for bisulfite sequencing.

According to the sequencing report from Sangon Biotech., the genomic DNA sample was first treated with sodium bisulfite and then purified according to the protocols of EpiTect® Bisulfite kit. PCR amplification of the bisulfite-treated genomic DNA was performed in 50 μL aqueous solution containing 0.2 mM dNTPs, 0.4 μM each of primer F (AAATCCGACATAATAACTAATAAACAAC) and primer B (GCGGTTAGTTTTGTATTGTAGGAG), PCR buffer (10 mM Tris-HCl, 50 mM KCl, 1.5 mM $MgCl_2$, pH 8.8), and 4 U of Pfu DNA Polymerase in a thermocycler with the following procedures: 98 °C for 4 min, followed by 20 cycles of 94 °C for 45 s, 66 °C for 45 s, and 72 °C for 1 min, and another 20 cycles of 94 °C for 45 s, 56 °C for 45 s, and 72 °C for 1 min, finally incubated at 72 °C for 8 min. The PCR product was finally sequenced with an ABI 3730xl DNA analyser (Applied Biosystems) by Sangon Biotech. (Shanghai, China).

The bisulfite sequencing result shown in Fig. S7 indicates that the sequence at the detected methylation site was GCGC, demonstrating that the cytosines in this target site are all methylated in the extracted genomic DNA from HCT116 cells.

9. The plots between POI values in the real-time fluorescence curves and logarithm (lg) of the target M' concentrations

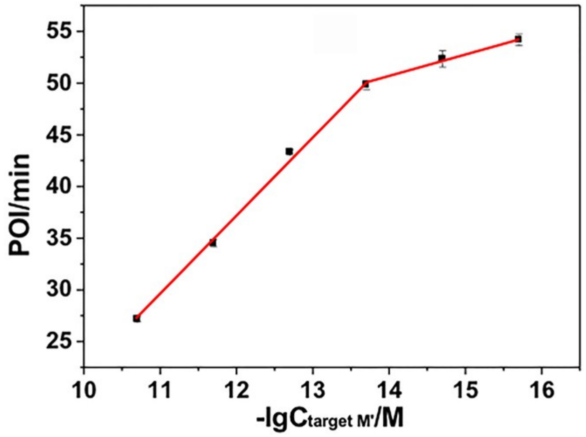

Fig. S8. The plots between POI values in the real-time fluorescence curves in Fig. 6 and logarithm (lg) of the target M' concentrations. Error bars are estimated from the standard deviation of three replicate measurements at each data point.
